# Pankreastransplantation – Klinik, Technik und histologische Beurteilung

**DOI:** 10.1007/s00292-021-00982-1

**Published:** 2021-08-20

**Authors:** Maike Büttner-Herold, Kerstin Amann, Frederick Pfister, Andrea Tannapfel, Marina Maslova, Andreas Wunsch, Nina Pillokeit, Richard Viebahn, Peter Schenker

**Affiliations:** 1 Abt. Nephropathologie, Pathologisches Institut, Universitätsklinikum Erlangen, Krankenhausstr. 8–10, 91054 Erlangen, Deutschland; 2 Institut für Pathologie, Ruhr-Universität Bochum, Bochum, Deutschland; 3 Institut für Diagnostische und Interventionelle Radiologie, Neuroradiologie und Nuklearmedizin, Universitätsklinikum Knappschaftskrankenhaus Bochum, Ruhr-Universität Bochum, Bochum, Deutschland; 4 Chirurgische Klinik, Universitätsklinikum Knappschaftskrankenhaus Bochum, Ruhr-Universität Bochum, Bochum, Deutschland

**Keywords:** Biopsie, Diabetes mellitus, Duodenum, Histologie, Nierentransplantation, Biopsy, Diabetes mellitus, Duodenum, Histology, Kidney transplantation

## Abstract

**Hintergrund:**

Die Pankreastransplantation wird in Deutschland nur in wenigen ausgewählten Zentren durchgeführt, üblicherweise in Kombination mit einer Niere. Die Kenntnis von Indikationen und Techniken der Transplantation selbst als auch der histopathologischen Abstoßungsdiagnostik mittels Pankreas- oder selten Duodenalbiopsie ist nicht sehr weit verbreitet.

**Ziel der Arbeit:**

Darstellung der Entwicklung und des aktuellen Stands der Pankreas-Nieren-Transplantation in Deutschland anhand der Erfahrungen des größten deutschen Zentrums und Analyse der Ergebnisse der zwischen 06/2017 und 12/2020 durchgeführten Abstoßungsbiopsien inklusive ausführlicher Darstellung und Bebilderung der verschiedenen Abstoßungskategorien

**Material und Methode:**

Es wurde eine ausführliche Literaturrecherche zur Historie, Technik und Indikation der Pankreastransplantation durchgeführt und die technischen Besonderheiten und Erfahrungen am Bochumer Zentrum, insbesondere auch die Komplikationen, im internationalen Vergleich dargestellt. Desweiteren wurden alle im Zeitraum zwischen 06/2017 und 12/2020 durchgeführten Pankreas- oder Duodenalbiopsien, die zur Abstoßungsdiagnostik nach Erlangen gegangen waren und mittels der Banff-Klassifikation standardisiert bearbeitet wurden, anhand der vorliegenden Befunde ausgewertet und zusammenfassend dargestellt. Zum besseren Verständnis wurden die wichtigsten histologischen Entitäten bildhaft dargestellt und differenzialdiagnostische Aspekte diskutiert.

**Ergebnisse:**

Insgesamt wurden 93 Pankreastransplantat- und 3 Duodenalbiopsien untersucht. In 32 Pankreasbiopsien, d.h. 34,4 %, war kein diagnostisch verwertbares Material enthalten. Bei den verbliebenen 61 Pankreasbiopsien fanden sich bei 24,6 % keine Abstoßungszeichen, 62,3 % eine akute T-Zell-vermittelte Abstoßungsreaktion (TCMR) und 8,2 % Hinweise auf eine aktive Antikörper-vermittelte Abstoßung (ABMR). Ein akuter Azinuszellschaden wurde in 59 % der Biopsien gesehen, eine Pankreatitis in 8,2 % und eine Allograft-Fibrose in immerhin 54,1 %. Die Calcineurin-Inhibitor (CNI) Toxizität war mit 4,9% eher selten.

**Schlussfolgerung:**

Die Pankreas-Nieren-Transplantation inklusive der Biopsie des transplantierten Pankreas oder in seltenen Fällen auch des Spenderduodenums mit anschließender standardisierter Beurteilung entsprechend der aktuellen international gültigen Banff-Klassifikation der Pankreasabstoßung und der Empfehlungen zur Beurteilung von Duodenalbiopsien hat ihren festen Stellenwert in der Behandlung von Diabetikern.

## Abstract (en)

**Background:**

In Germany pancreas transplants are performed in only a few selected and specialized centres, usually combined with a kidney transplant. Knowlegde of the indications for and techniques of transplantation as well as of the histopathological assessment for rejection in pancreas and duodenal biopsies is not very widespread.

**Aim:**

To give an overview of the development and status quo in pancreas-kidney-transplantation in Germany summarizing the experience of the largest German pancreas transplant centre and to give a résumé of the results of histological diagnoses of biopsy specimens submitted between 06/2017 and 12/2020. Moreover, a detailed description and illustration of histological findings is included.

**Material and Methods:**

A thorough literature search for aspects of the history, technique and indication for pancreas transplantation was performed and discussed in the context of the local experience and technical particularities specific for the transplant centre in Bochum. The occurrence of complications was compared with international reports. Results of pancreas and duodenal biopsies submitted to Erlangen between 06/2017 and 12/2020 for histological evaluation, which were evaluated according to the Banff classification, were summarized. For a better understanding key histological findings of pancreas rejection and differential diagnoses were illustrated and discussed.

**Results:**

A total of 93 pancreas transplant specimens and 3 duodenal biopsies were included. 34.4% of pancreas specimens did not contain representative material for a diagnosis. In the remaining 61 biopsies 24.6% showed no rejection, 62.3% were diagnosed with acute T-cell mediated rejection (TCMR) and 8.2% with signs suspicious of antibody-mediated rejection (ABMR). Acute acinary epithelial injury was seen in 59%, pancreatitis in 8.2% and allograft fibrosis was reported in as many as 54.1%. Calcineurin-inhibitor toxicity was discussed in only 4.9%.

**Conclusion:**

Pancreas-kidney-transplantation and standardized histological assessment of the transplanted pancreas or rarely duodenum with reporting according to the updated Banff classification of pancreas transplants or previous reports of duodenal rejection are important mainstays in the management of patients with diabetes.

Seit Durchführung der ersten Pankreastransplantation (PTX) 1966 durch William Kelly und Richard Lillehei an der University von Minnesota wurden bis 2017 weltweit etwa 50.000 Pankreastransplantationen durchgeführt [1, 2]. Im Jahr 1979 erfolgte von Walter Land in München-Großhadern die erste PTX in Deutschland als Pankreassegmenttransplantation mit Verschluss des Pankreasganges [3]. Seitdem hat die PTX eine bemerkenswerte Entwicklung durchgemacht.

Durch Modifikationen des operativen Verfahrens, Fortschritte in der Organkonservierung und Intensivmedizin sowie die Verfügbarkeit besserer Immunsuppressiva hat sich die PTX von einem experimentellen Verfahren zu einer akzeptierten und erfolgreichen Therapieoption des Diabetes mellitus entwickelt. Die kombinierte Pankreas‑/Nierentransplantation (PNTX) stellt inzwischen die Therapie der Wahl für niereninsuffiziente Typ-I-Diabetiker dar [4]. Lag das 1‑Jahres-Überleben von Patienten und Pankreastransplantat nach kombinierter PNTX 1980 noch bei 67 % bzw. 21 %, so werden aktuell nach einem Jahr Werte von 98 % Patienten- und 89 % Pankreastransplantatüberleben angegeben [2]. Die erfolgreiche PTX führt neben einer signifikanten Verbesserung der Lebensqualität zu einer nahezu vollständigen Normalisierung des Glukosestoffwechsels [5–7]. Daraus resultiert eine Reduktion der kardiovaskulären Mortalität und somit ein Vorteil für das Langzeitüberleben der Patienten [8, 9]. Das Fortschreiten der diabetischen Spätschäden, Neuropathie, Retinopathie, Nephropathie und Mikroangiopathie wird vermindert. Demgegenüber steht eine deutlich erhöhte Morbidität und Mortalität in den ersten Monaten nach Transplantation im Vergleich mit isolierter Nierentransplantation [10]. Nach wie vor ist die PTX mit der höchsten Komplikationsrate aller soliden Organtransplantationen behaftet. Relaparotomien, Infektionen und Rejektionen sind signifikant häufiger, die perioperative Mortalität ist höher und die Krankenhausverweildauer länger als bei einer alleinigen Nierentransplantation (NTX). PTX werden aktuell lediglich an wenigen Zentren in nennenswertem Umfang durchgeführt und erreichen im Vergleich zu den sonstigen Organtransplantationen nur geringe absolute Zahlen. In Deutschland wurden im Jahr 2019 an 23 Zentren insgesamt 94 Bauchspeicheldrüsen transplantiert. In 16 der Zentren lag die jährliche Fallzahl bei ≤ 3 PTX [11]. Die meisten PTX erfolgen zusammen mit einer Niere des gleichen Spenders als simultane PNTX („simultaneous pancreas-kidney“, SPK). Weitere Transplantationsvarianten stellen die selten durchgeführte alleinige PTX („pancreas transplant alone“, PTA) und die PTX nach bereits erfolgter NTX („pancreas after kidney“, PAK) dar.

## Simultane Pankreas‑/Nierentransplantation (SPK)

Die klassische Indikation zur simultanen PNTX stellt der juvenile Typ I‑Diabetes mit terminaler Niereninsuffizienz dar. Eine kombinierte PNTX ist jedoch auch im Stadium der präterminalen Niereninsuffizienz möglich. So kann ein Patient ab Stadium 4 der chronischen Niereninsuffizienz (glomeruläre Filtrationsrate [GFR], < 30 ml/min) in die Warteliste für eine kombinierte PNTX aufgenommen werden. Diese präemptive Transplantation führt zu einer Reduktion der perioperativen Mortalität und verbessert das Langzeitüberleben der Patienten erheblich. In Europa erfolgt ein Großteil (89 %) der PTX zusammen mit einer Nierentransplantation [12]. Dabei stammen beide Organe vom selben Organspender.

## Alleinige Pankreastransplantation (PTA)

In seltenen Fällen kann bei Patienten mit extrem instabilem Diabetes mellitus Typ I (sog. Brittle-Diabetes) die Indikation zur alleinigen PTX gestellt werden. Hypoglykämie-Wahrnehmungsstörungen, das Vorliegen einer subkutanen Insulinresistenz, aber auch ein frühes Auftreten und rasches Fortschreiten diabetischer Spätschäden können eine alleinige PTX rechtfertigen. In diesen Fällen sollte die Nierenfunktion aufgrund der zu erwartenden Nephrotoxizität der Immunsuppressiva nicht oder nur gering beeinträchtigt sein. Trotz Verbesserungen der Ergebnisse ist die alleinige PTX keine prinzipielle Alternative zu konservativen Therapiemöglichkeiten. Die Indikation sollte streng gestellt werden und nur interdisziplinär, z. B. im Rahmen der Transplantationskonferenz, nachdem wichtige Alternativen (z. B. eine Insulinpumpentherapie) ausgeschöpft und selbstinduzierte Hypoglykämien bei psychischen Störungen ausgeschlossen wurden [13]. Es muss eine sorgfältige Abwägung des Operationsrisikos und der Nebenwirkungen der immunsuppressiven Therapie gegenüber den zu erwartenden Vorteilen einer Transplantation erfolgen.

## Pankreastransplantation nach erfolgter Nierentransplantation (PAK)

Nach stattgehabter Leichennieren- oder Lebendnierentransplantation eines Typ-I-Diabetikers kann eine PTX erfolgen. Bei dieser Art der Transplantation fehlt die immunologische Identität der Transplantate. Von Vorteil ist jedoch, dass die Immunsuppression zum Zeitpunkt der PTX bereits besteht. Weitere Vorteile dieser Transplantationsform sind eine reduzierte Wartezeit sowie eine geringere Mortalität im Vergleich zur simultanen PNTX [14].

## Indikationen zur Pankreastransplantation

Entsprechend der aktuellen Richtlinie für die Wartelistenführung und die Organvermittlung zur PTX und kombinierten PNTX müssen folgende Kriterien für die Aufnahme in die Warteliste erfüllt werden [15]:

Vorhandensein von Autoantikörpern gegen Glutamatdecarboxylase (GAD) und/oder Inselzellen (ICA) und/oder Tyrosinphosphatase 2 (IA-2) und/oder Zinktransporter 8 (ZnT8) und/oder Insulin (IAA).

Der Autoantikörpernachweis von GAD, IA‑2, ICA und ZnT8 kann zum Zeitpunkt der Listung oder in der Vergangenheit erfolgt sein. Der positive Befund für IAA ist nur dann akzeptabel, wenn das Nachweisdatum vor Beginn der Insulintherapie liegt. Daher muss für den Nachweis der IAA das Datum der Testung und der Beginn der Insulintherapie an Eurotransplant übermittelt werden. Können keine Autoantikörper detektiert werden, muss eine Betazelldefizienz nachgewiesen werden. Diese wird in der aktuellen Richtlinie definiert als:

- C‑Peptid vor Stimulation < 0,5 ng/ml mit einem Anstieg nach Stimulation von < 20 %, wenn parallel zu diesem Messzeitpunkt kein Blutzuckerwert vorliegt oder
- C‑Peptid vor Stimulation < 0,5 ng/ml mit einem gleichzeitig erhobenen Blutzuckerwert ≥ 70 mg/dl (bzw. ≥ 3,9 mmol/l) oder
- C‑Peptid nach Stimulation < 0,8 ng/ml, mit einem gleichzeitig einhergehenden Blutzuckeranstieg auf ≥ 100 mg/dl (bzw. ≥ 5,6 mmol/l).

Als Stimulationstests werden ein oraler Glukosetoleranztest (OGTT), ein Mixed-Meal-Toleranztest (MMTT) oder ein intravenöser oder subkutaner Glukagontest akzeptiert. Zusätzlich können bedrohliche und rasch fortschreitende diabetische Spätfolgen, das Syndrom der unbemerkten schweren Hypoglykämie, ein exzessiver Insulinbedarf oder fehlende Applikationswege für Insulin bei subkutaner Insulinresistenz eine Indikation zur isolierten PTX darstellen. Über die mögliche Aufnahme in die Warteliste entscheidet dann in jedem Einzelfall eine Auditgruppe bei der Vermittlungsstelle (Eurotransplant Pancreas Advisory Committee, EPAC).

Die PTX bei Typ-2-Diabetikern wird weiterhin kontrovers diskutiert. Der Anteil der pankreastransplantierten Typ-2-Diabetiker liegt weltweit zwischen 1 % und 8 %, ist aber in den letzten Jahren kontinuierlich angestiegen [2]. Schlanke Typ-2-Diabetiker mit geringem Insulinbedarf und Niereninsuffizienz sowie Patienten mit Typ-MODY-Diabetes („maturity onset diabetes of the young“) profitieren in ähnlicher Weise von einer kombinierten PNTX wie Typ-1-Diabetiker [16]. Auch hier entscheidet das EPAC über die Aufnahme auf die Warteliste. Kontraindikationen für eine PTX stellen eine bestehende Malignomerkrankung, eine nicht sanierte akute oder chronische Infektion sowie eine schwere psychische Störung und Non-Adhärenz dar. Ebenfalls können ausgeprägte kardiovaskuläre Erkrankungen, soweit nicht therapierbar, eine Kontraindikation darstellen. Bei Patienten mit einem Body-Mass-Index (BMI) > 30 kg/m^2^ sollte nur in Ausnahmefällen eine PTX durchgeführt werden. Einerseits besteht eine Assoziation zwischen Übergewicht und der perioperativen Morbidität und Mortalität, andererseits kann der Transplantationserfolg mit Erreichen einer Insulinunabhängigkeit und Normoglykämie durch das bestehende Übergewicht gefährdet werden. Das Patientenalter allein stellt keine Kontraindikation zur PTX dar. Waren in den letzten Jahrzehnten noch viele Zentren sehr zurückhaltend bei der PTX über 50-jähriger Patienten, so konnte in den letzten Jahren, insbesondere in High-volume-Zentren, gezeigt werden, dass auch bei älteren Patienten durchaus gute Ergebnisse nach einer PTX erzielt werden können [17, 18]. Kardiovaskuläre Erkrankungen stellen mit Abstand den größten Komorbiditätsfaktor bei Patienten mit Diabetes mellitus Typ I und Niereninsuffizienz dar. Potenzielle Pankreastransplantatempfänger mit kardialer Anamnese, Auffälligkeiten in der nichtinvasiven Diagnostik (Echokardiografie, Myokardszintigrafie), einem Lebensalter > 50 Jahre oder bereits bestehender Dialysepflichtigkeit sollten vor einer PTX immer einer Koronarangiografie unterzogen werden. Ebenso sorgfältig sollten die peripheren Gefäßverhältnisse, insbesondere die Iliakalgefäße, hinsichtlich ihrer Anastomosierungsfähigkeit untersucht werden.

## Spenderkriterien

Die Spenderkriterien sowie eine optimale Organentnahme und Organkonservierung sind mitentscheidend für den Erfolg einer PTX. Eine Vielzahl von Spenderparametern wurde hinsichtlich ihrer Bedeutung für das Ergebnis nach PTX untersucht. Wesentliche Faktoren sind das Spenderalter, BMI, Todesursache (traumatisch oder kardiovaskulär), Verweildauer auf der Intensivstation, erfolgte Reanimation, hypotensive Phasen und der Einsatz von Katecholaminen. Des Weiteren fließen Laborparameter wie Serumamylase und Serumlipase, Vorliegen einer Hypernatriämie, Blutzucker und Serumkreatinin in die Entscheidung über die Transplantabilität eines Spenderpankreas ein [19, 20]. Die Dauer der Ischämiezeit beeinflusst die Rate und Intensität von Pankreatitiden und somit die Funktionsrate nach Transplantation, während der Einfluss verschiedener Konservierungslösungen kontrovers diskutiert wird. Neben dem Vorliegen von Malignomen und Infektionskrankheiten gelten ein Diabetes mellitus, eine akute oder chronische Pankreatitis, vorausgegangene chirurgische Eingriffe am Pankreas und ein höhergradiges Pankreastrauma als Kontraindikationen beim Spender. Von einigen Autoren werden ein chronischer Alkoholabusus, eine intraabdominelle Sepsis sowie ein BMI > 35 kg/m^2^ ebenfalls als Kontraindikationen angesehen [20]. Viele Transplantationschirurgen akzeptieren keine Spenderpankreata mit Kalzifikationen, fortgeschrittener Organfibrose, infiltrativen Verfettungen, deutlichem Ödem sowie einer ausgeprägten Atherosklerose der Viszeral- und Beckenarterien des Spenders. Ebenso von Bedeutung ist die zu palpierende Konsistenz des Organs. Dabei ist die intraoperative Beurteilung des Pankreas durch einen erfahrenen Transplantationschirurgen von wesentlicher Bedeutung. Diese Kriterien erscheinen etwas subjektiv, sind jedoch in der Allokationsrealität Laborwerten und demografischen Daten des Spenders überlegen. Im Vergleich mit der Akzeptanz anderer Organe zur Transplantation ist die Ablehnungsrate potenzieller Pankreasspender sowohl in Europa als auch in den USA hoch. So wurden z. B. im Jahr 2019 nur 21 % der bei Eurotransplant gemeldeten Spenderpankreata transplantiert [21].

## Technik der Pankreastransplantation

**Figure 1:**
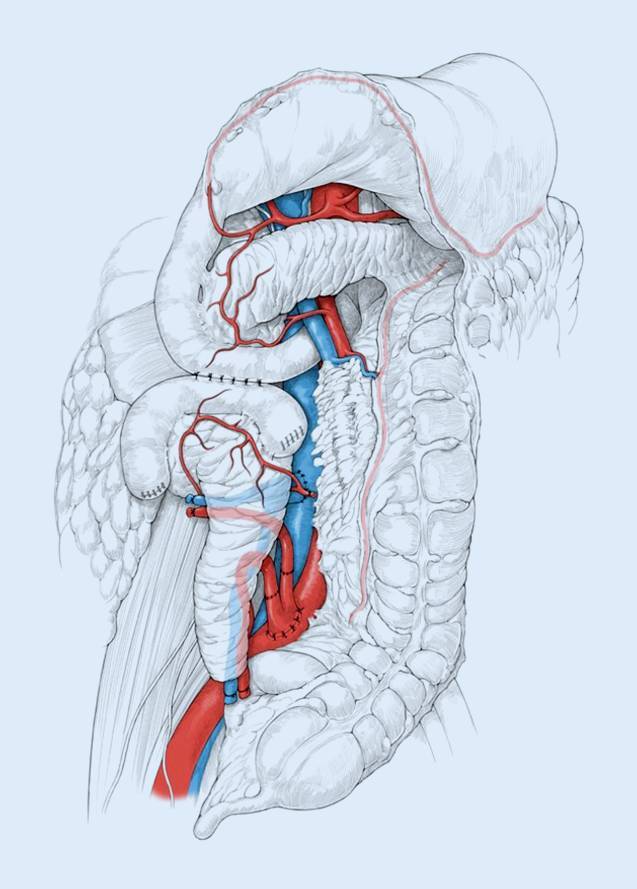

Die klinische PTX unterlag zahlreichen Veränderungen und Modifikationen hinsichtlich der chirurgischen Technik. Auch heute werden diverse Techniken nebeneinander angewendet, ohne dass letztlich die Überlegenheit der einen oder anderen Methode nachgewiesen werden konnte. Allein die Tatsache, dass in einigen Zentren parallel verschiedene Techniken Verwendung finden, verdeutlicht diese Situation. Nach ihrer Einführung war die PTX mit einer sehr hohen peri- und postoperativen Morbidität und Mortalität behaftet, sodass sie viele Jahre als experimentelles Verfahren angesehen wurde. Vordergründig für die schlechten Ergebnisse zu dieser Zeit war das ungenügende Management der exokrinen Pankreassekretion. Dieses ist auch heute noch von zentraler Bedeutung, da die Freisetzung von aktivierten Pankreasenzymen, ähnlich wie bei der akuten Pankreatitis, zu gravierenden Gewebeschädigungen führen kann. Erst mit der von Sollinger 1983 [22] entwickelten Blasendrainagetechnik und der Einführung der Pankreasduodenaltransplantation durch Nghiem und Corry 1987 ist die PTX im Hinblick auf das Auftreten von Nahtinsuffizienzen und Pankreasfisteln sicherer geworden [23]. Bei dieser Technik wird das gesamte Spenderpankreas mit einem blindverschlossenen, kurzen Duodenalsegment transplantiert. Das exokrine Sekret wird dabei über eine in Seit-zu-Seit-Technik angelegte Duodenozystostomie in die Harnblase abgeleitet. Dieses Verfahren erlaubte durch Bestimmung von Amylase und Lipase im Urin ein Transplantatmonitoring, war jedoch durch das Auftreten schwerer metabolischer Azidosen, Dehydratationen, Harnblasen- und Harnröhrenentzündungen sowie Refluxpankreatitiden mit vielen Komplikationen behaftet. Mit der Verfügbarkeit einer besseren Immunsuppression und der damit verbundenen Reduktion immunologisch bedingter Transplantatverluste hat sich seit Mitte der 1990er-Jahre in den meisten Transplantationszentren die enterale Drainage in den Dünndarm durchgesetzt. Hierbei wird das exokrine Pankreassekret durch eine Seit-zu-Seit-Duodenojejunostomie oder eine Duodenoduodenostomie in den Darm abgeleitet. Auch Rekonstruktionen über eine nach Roux‑Y ausgeschaltete Dünndarmschlinge finden Anwendung. In den letzten Jahren wird von einigen Zentren eine retroperitoneale Platzierung des Pankreastransplantates favorisiert [24, 25]. Die endokrine Drainage oder venöse Ableitung des Pankreastransplantates kann systemisch-venös in die V. cava inferior oder portal-venös in die V. mesenterica superior erfolgen. Ob das technisch anspruchsvollere, aber physiologischere Verfahren der portal-venösen Drainage metabolische Vorteile hat, ist bisher nicht bewiesen. Beide Verfahren führen zu vergleichbaren Ergebnissen. Seit 2007 bevorzugen wir die retroperitoneale Positionierung des Transplantates unter Anlage einer Seit-zu-Seit-Duodenoduodenostomie mit portal-venöser oder systemisch-venöser Anastomosierung (Abb. 1). Der Vorteil der Duodenoduodenostomie besteht darin, dass sowohl die Dünndarmanastomose als auch der Pankreaskopf des Transplantates endoskopisch erreicht werden können. Somit wird es möglich, endoskopisch Biopsien zur Abstoßungsdiagnostik zu gewinnen. Des Weiteren kann im Falle einer intestinalen Blutung im Anastomosenbereich eine einfache endoskopische Intervention zur Blutstillung erfolgen. Mögliche Nachteile ergeben sich im Falle einer Anastomoseninsuffizienz oder eines Transplantatverlustes, da die resultierende Leckage im Bereich des Duodenums chirurgisch schwieriger zu versorgen ist. Neben vielen weiteren Vorteilen der retroperitonealen Positionierung ist das Pankreastransplantat besser sonografisch darstellbar und einfach perkutan zu punktieren.

## Komplikationen nach Pankreastransplantation (PTX)

### Transplantatthrombose

Die Pankreastransplantatthrombose ist nach wie vor die häufigste Ursache für einen frühen Verlust des Pankreastransplantates. Bei einem plötzlichen Blutzuckeranstieg in der Frühphase nach Transplantation muss nach Ausschluss anderer Ursachen immer an eine Perfusionsstörung des Pankreastransplantates gedacht werden. Der Nachweis erfolgt in der Regel durch eine CT oder MRT des Abdomens. Die farbcodierte Duplexsonografie kann erste Hinweise auf Perfusionsstörungen ergeben. Im eigenen Zentrum hat sich in den letzten Jahren die kontrastmittelunterstützte Sonografie (CEUS) zum Perfusionsnachweis als extrem hilfreich erwiesen, da diese sofort auf Station im Patientenbett durchgeführt werden kann und kein potenziell nephrotoxisches Kontrastmittel appliziert werden muss. Bei Nachweis einer Thrombose im Bereich der Pfortader oder Milzvene sowie Thrombosen im Bereich des arteriellen Zuflusses (Y-Graft) ist eine sofortige Relaparotomie durchzuführen. In einigen Fällen ist es dabei gelungen, erfolgreich eine Thrombektomie durchzuführen und die Organfunktion zu erhalten. Auch Erfolge bei Lysetherapien wurden berichtet. Häufig bleibt einem jedoch nur die Entfernung des thrombosierten Transplantates übrig.

### Transplantatpankreatitis

Das Entstehen einer Transplantatpankreatitis ist oftmals schon intraoperativ nach Reperfusion zu bemerken. Aber auch nach der Reperfusion unauffällig anmutende Transplantate können im Verlauf schwerwiegende Formen einer Pankreatitis entwickeln. In der Diagnostik ergänzen sich klinischer Befund, Laboruntersuchungen und Bildgebung. Bei der klinischen Untersuchung finden sich meist rechtsseitig betonte Bauchschmerzen ggf. mit Peritonismus. Zusätzlich können das Entstehen eines paralytischen Ileus, Fieber sowie eine Transplantatdysfunktion auf eine Pankreatitis hinweisen. Laborchemisch besteht häufig eine deutliche CRP-Erhöhung, die mit einem Anstieg von Serumamylase und Serumlipase assoziiert sein kann. In der Bildgebung können alle Formen einer Pankreatitis imponieren. Diese reichen von einer leichten ödematösen Form bis hin zur nekrotisierenden Pankreatitis mit Perfusionsausfällen. Bei schweren, lebensbedrohlichen Verlaufsformen einer Transplantatpankreatitis kann die partielle oder komplette Entfernung des Transplantates notwendig werden, auch wenn eine gute Funktion vorliegt.

### Blutung

Bei Blutungskomplikationen nach einer PTX muss zwischen intraluminaler, intestinaler Blutung im Bereich der Anastomose und einer Blutung im Bauchraum unterschieden werden. Die Möglichkeit der einfachen endoskopischen Blutstillung im Bereich der Duodenoduodenostomie ist einer der großen Vorteile dieser Anastomosierungstechnik. Aber auch bei Anastomosen im Bereich der 1. und 2. Jejunalschlinge und intraperitonealer Positionierung des Pankreastransplantates ist über eine Push-Intestinoskopie die Anastomose zu erreichen, wobei sich dies technisch schwieriger gestaltet. Intraabdominelle Blutungen nach PTX sind kein seltenes Ereignis, zumal ein Großteil der Patienten eine postoperative Heparintherapie erhält. Kleinere Blutungen können oftmals konservativ beherrscht werden, indem die Antikoagulantientherapie pausiert wird und plasmatische Gerinnungsfaktoren optimiert werden. Hämodynamisch relevante Blutungen und ein rezidivierender Transfusionsbedarf zeigen die Indikation zur Relaparotomie an. Das Spektrum solcher Nachblutungen reicht von kleinen diffusen Blutungen aus dem Transplantatpankreas (häufig an der Mesenterialwurzel) oder kleineren Blutungen an den peritonealen Inzisionslinien bis hin zu Katastrophenblutungen aus der unteren Hohlvene und der Beckenarterie. Bei Arrosionsblutungen im Bereich der Beckenarterie kann in manchen Fällen das endovaskuläre Stenting der Defektzone die rettende Maßnahme für den Patienten sein.

### Akute Transplantatabstoßung

Ein Pankreastransplantat kann Ziel der allogenen Immunität (Abstoßung) und/oder der Autoimmunität (Rekurrenz der Grunderkrankung) sein. Amylase- und Lipaseerhöhung im Serum sowie ein Anstieg der Blutzuckerwerte sind häufig die einzigen, aber unspezifischen Zeichen einer Abstoßung. Im Falle einer kombinierten PNTX ist die histologische Diagnose aus dem Nierentransplantat oftmals ausschlaggebend. Es konnte aber auch gezeigt werden, dass eine nicht unerhebliche Diskordanz beim Auftreten von Rejektionen und deren Schweregrad in zeitgleich entnommenen Biopsien aus Pankreas- und Nierentransplantat besteht. Uva et al. [26] beschrieben lediglich in 40 % der Fälle mit Abstoßungen ein zeitgleiches Auftreten der Rejektion in beiden Organen. In einer Studie von Parajuli et al. [27] lag die Diskordanzrate für Abstoßungen bei 37,5 %. Bei den 62,5 % der Fälle mit konkordanten Befunden in beiden Organen wurden wiederum unterschiedliche Abstoßungstypen gefunden. Im eigenen Zentrum konnten wir nach zeitgleicher Punktion von Pankreas und Niere eine Konkordanz für das Auftreten bzw. Ausschluss einer Rejektion in beiden Organen von 68,4 % feststellen. Bei einer isolierten PTX oder einer PAK-Transplantation, bei welcher die Spenderorgane nicht HLA-identisch sind, spielt die Pankreasbiopsie eine noch wichtigere Rolle. Die histopathologische Begutachtung ist aufwendig und schwierig. Aufgrund der an sich schon geringen Fallzahlen der PTX und der noch seltener durchgeführten Pankreastransplantatbiopsien ist vielerorts nur begrenzte oder keine Erfahrung vorhanden, sodass wie bei anderen Spezialuntersuchungen die Biopsien meist überregional verschickt und begutachtet werden. Für die Beurteilung von Pankreastransplantatbiopsien existiert, ähnlich wie bei der NTX, seit vielen Jahren ein international anerkanntes und ständig aktualisiertes Banff-Schema zur Graduierung der Abstoßung (Tab. 1; [28–30]).

**Table 1:** 

*1. Normal*
*2. Unklar*
*3. Akute T‑Zell-vermittelte Abstoßung (TCMR)*
*Grad I/mild:* aktive septale Entzündung mit Venulitis und/oder Duktitis und/oder fokale azinäre Entzündung (≤ 2 Foci pro Läppchen) ohne oder nur mit minimalem Azinuszellschaden
*Grad II/moderat* (erfordert eine Abgrenzung zur ABMR): multifokale (aber nicht konfluente oder diffuse) azinäre Entzündung (≥ 3 Foci pro Läppchen) mit vereinzeltem, individuellem Azinuszellschaden und/oder milde intimale Arteriitis (< 25 % des Lumens)
*Grad III/schwer* (erfordert eine Abgrenzung zur ABMR): diffuse azinäre Entzündung mit fokaler oder diffuser, multizellulärer/konfluierender Azinuszellnekrose und/oder moderate oder schwere intimale Arteriitis (> 25 % des Lumens) und/oder transmurale Entzündung – nekrotisierende Arteriitis
*4. Akute/aktive Antikörper-vermittelter Abstoßung (ABMR)*
a) morphologischer Nachweis eines akuten Gewebeschadens
Grad I (milde akute ABMR): Gut erhaltene Architektur, milde interazinäre Monozyten‑/Makrophagen- oder gemischte (Monozyten‑/Makrophagen/neutrophile) Infiltrate mit seltenem Azinuszellschaden (Schwellung, Nekrose)
Grad II (moderate akute ABMR): Insgesamt erhaltene Architektur mit interazinären Monozyten-Makrophagen-Infiltraten oder gemischte (Monozyten-/Makrophagen/neutrophile) Infiltrate, Dilatation der Kapillaren, interazinäre Kapillaritis, intimale Arteriitis, Stauung, multizellulärer Azinuszellverlust und Erythrozytenextravasation
Grad III (schwere akute ABMR): Architekturstörung, verstreute Entzündungsinfiltrate bei interstitiellen Einblutungen, multifokaler oder konfluierender Parenchymnekrose, arterieller oder venöser Gefäßwandnekrose, transmuraler/nekrotisierender Arteriitis und Thrombose (in Abwesenheit anderer offensichtlicher Ursachen)
b) C4d-Positivität der interazinären Kapillaren (IAC) ≥ 1 % der Azinusläppchenoberfläche
c) Bestätigte donorspezifische Antikörper (DSA)
Abschließende Einordnung:
1 von 3 diagnostischen Kriterien: benötigt Ausschluss einer ABMR
2 von 3 diagnostischen Kriterien: ABMR muss erwogen werden
3 von 3 diagnostischen Kriterien: definitive Diagnose einer ABMR
*5. Chronisch-aktive ABMR*
Kategorie 3 und/oder 4 mit chronischer Arteriopathie und/oder Kategorie 6. Spezifikation, ob TCMR, ABMR oder gemischt
*6. Chronische Arteriopathie* Fibrointimale arterielle Verbreitung mit Lumeneinengung: inaktiv: fibrointimal aktiv: Infiltration der subintimalen fibrösen Proliferation durch mononukleäre Zellen (T-Zellen und Makrophagen)
Grad 0: keine Lumeneinengung
Grad 1: mild, ≤ 25 % Lumeneinengung
Grad 2: moderat, 26–50 % Lumeneinengung
Grad 3: schwer, ≥ 50 % Lumeneinengung
*7. Chronische Transplantatfibrose*
Grad I (milde Transplantatfibrose): < 30 %
Grad II (moderate Transplantatfibrose): 30–60 %
Grad III (schwere Transplantatfibrose): > 60 %
*8. Inselpathologie:* Rekurrenz des autoimmunen Diabetes mellitus (Insulitis und/oder selektiver Betazellverlust), Amyloidablagerungen, Calcineurininhibitor-Toxizität der Inselzellen
*9. Andere histologische Veränderungen,* welche nicht einer akuten und/oder chronischen Abstoßung zugeordnet werden, wie z. B. CMV-Pankreatitis, posttransplantations-lymphoproliferative Erkrankung

Die Therapie der akuten Abstoßung eines Pankreastransplantates unterscheidet sich nicht wesentlich von der Abstoßungstherapie anderer transplantierter Organe. Bei milden akuten T‑zellulären Abstoßungen (Banff Grad I) erfolgt auch nach PTX eine Steroidbolustherapie mit z. B. 3 × 500 mg Prednisolon i.v. und eine Intensivierung der Basisimmunsuppression. Bei höhergradigen zellulären Abstoßungen und Rezidivabstoßungen erfolgen Therapien mit T‑Zell-depletierenden Antikörpern (ATG), bei humoraler Abstoßungskomponente auch in Kombination mit einer Anti-B-Zell-Therapie (z. B. Rituximab), Plasmapheresebehandlungen und die Gabe von intravenösen Immunglobulinen (IVIG) [31].

## Indikation und Technik der Pankreastransplantatbiopsie

### Indikationen

Nach einer PTX kann eine Biopsie aus verschiedenen Indikationen heraus notwendig werden. Neben der Durchführung von Indikationsbiopsien aufgrund einer Störung der Transplantatfunktion oder immunologischen Vorgängen werden von einigen Zentren auch sog. Protokollbiopsien, d. h. routinemäßige Entnahmen von Biopsien nach einem definierten Schema entnommen. Störungen der Pankreastransplantatfunktion äußern sich klinisch am häufigsten durch erhöhte Blutzuckerwerte. Diese können mit einem Anstieg von Serumamylase und Serumlipase assoziiert sein. Differenzialdiagnostisch müssen dabei verschiedene Ursachen in Betracht gezogen werden [31–33]: eine primäre Nichtfunktion oder verzögerte Funktionsaufnahme des Transplantates, eine Transplantatpankreatitis, eine Thrombose, eine akute oder chronische Rejektion, eine medikationsbedingte Hyperglykämie (Steroide, Tacrolimus), Infektionen, eine Rekurrenz der Grunderkrankung, eine Toxizität von Calcineurininhibitoren (CNI) oder die Manifestation eines Diabetes mellitus Typ 2. Einen Überblick über häufige Indikationen zur Biopsie des Pankreastransplantates gibt Tab. 2.

**Table 2:** 

Indikation	
Störungen der endokrinen Funktion	Hyperglykämie
	Gestörte Glukosetoleranz
	Erhöhter HbA1c-Wert
Störungen der exokrinen Funktion	Erhöhte Amylase- und Lipasewerte
Humorale Abstoßung	Donorspezifische Antikörper (DSA)
Rekurrenz der Grunderkrankung	Diabetes mellitus Typ 1 assoziierte Autoantikörper
Protokollbiopsien	Protokoll
Kontrollbiopsien	Nach z. B. stattgehabter Abstoßungstherapie

### Technik der Biopsieentnahme

**Figure 2:**
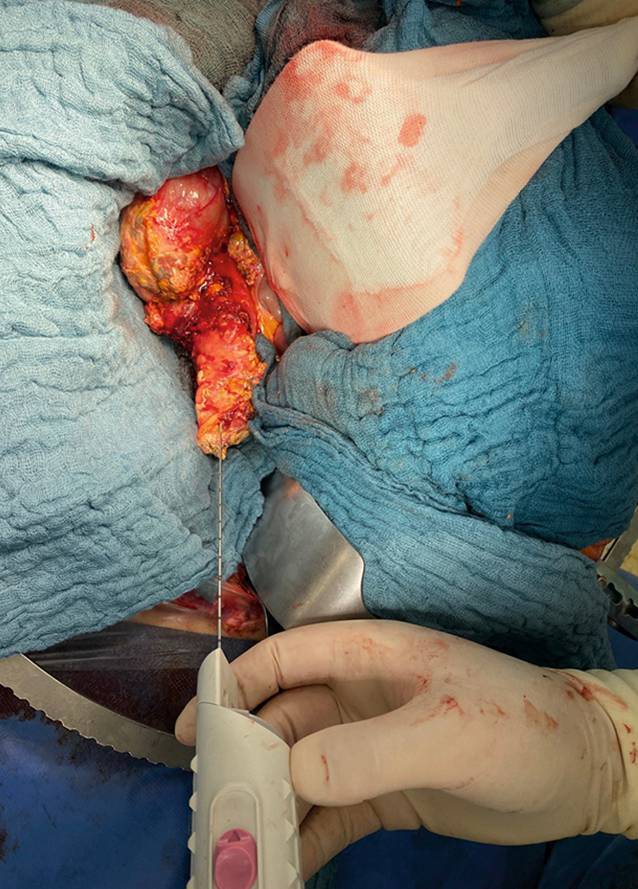

Die Pankreastransplantatbiopsie kann über verschiedene Zugangswege gewonnen werden. Dazu muss zwischen operativen, endoskopischen und perkutanen Techniken unterschieden werden. Bei den operativen Verfahren stellt die Laparotomie die invasivste Variante dar. Biopsien können jedoch im Rahmen von Laparotomien aus anderen Gründen einfach simultan als Punktions- oder Exzisionsbiopsie gewonnen werden (Abb. 2). Bei intraperitonealer Lage des Transplantates und damit häufiger Überlagerung des Transplantates von Dünndarmschlingen wird zunehmend die laparoskopisch assistierte Pankreasbiopsie angewendet. Limitiert wird das laparoskopische Vorgehen in einigen Fällen durch postoperative Verwachsungen, die eine Zugänglichkeit des Transplantates erschweren und das Risiko einer perioperativen Komplikation erhöhen [34]. Die früher häufig und heutzutage nur noch selten durchgeführte Blasendrainage des Pankreastransplantates, bei der das Spenderduodenum mit der Harnblase anastomosiert wird, ermöglicht eine einfache zystoskopische Biopsie aus dem Spenderduodenum oder Kopf des Pankreastransplantates [35]. Die histopathologische Aufarbeitung der Biopsien vom Spenderduodenum und Pankreastransplantat ergaben jedoch Diskrepanzen im Grad der Abstoßung. Hierbei konnte das Spenderduodenum unabhängig vom Pankreastransplantat eine Abstoßung aufweisen und umgekehrt [36]. Bei der heute hauptsächlich verwendeten Dünndarmdrainagetechnik ist die Entnahme einer Biopsie aus dem Spenderduodenum im Rahmen einer Ösophagogastroduodenoskopie oder Push-Intestinoskopie möglich [36, 37]. Auch hier konnten deutliche Diskordanzen beim histologischen Nachweis einer Abstoßung im Spenderduodenum und Pankreastransplantat beobachtet werden [35, 36]. Mittels Endosonografie lassen sich sowohl aus dem Spenderduodenum als auch aus dem Pankreastransplantat Feinnadelbiopsien entnehmen, wobei die gewonnene Gewebemenge oftmals eine aussagekräftige Diagnose nicht erlaubt.

**Figure 3:**
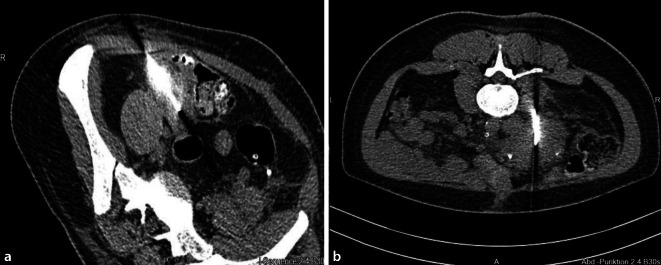

Im eigenen Zentrum hat sich im letzten Jahrzehnt die perkutane Pankreastransplantatbiopsie durchgesetzt. Die mit diesem Verfahren gewonnenen Gewebestanzen erlauben, ähnlich wie bei der Nierentransplantatbiopsie, eine aussagekräftige histopathologische Diagnosestellung. Die perkutane Biopsie erfolgt in der Regel in Lokalanästhesie unter sonografischer oder CT-gesteuerter Kontrolle. Bei beiden Methoden wird das Parenchym des Pankreastransplantates mit einer automatisierten 16G- oder 18G-Tru-Cut®-Nadel punktiert. Dabei werden 2–3 Punktionszylinder entnommen. Je nach Anatomie bieten sich hierbei verschiedene Zugangswege an (Abb. 3). Die Schwierigkeit in der genauen Punktionslokalisation liegt bei der CT darin, dass diese nativ durchgeführt wird und sowohl das Parenchym als auch die Transplantatgefäße schlecht visualisiert werden. Im Vorfeld angefertigte CT- oder MRT-Bilder sind daher hilfreich. In der Literatur finden sich Raten von bis zu 30 %, in denen bioptisch kein Pankreasgewebe gewonnen werden konnte [38]. In den letzten Jahren konnten wir im eigenen Vorgehen unter Nutzung der kontrastmittelgestützten Sonografie (CEUS) die Trefferquote erhöhen. In jedem Fall ist die Zusammenarbeit zwischen Transplantationschirurgen und Radiologen essenziell, um die Treffsicherheit zu erhöhen und die Komplikationsrate gering zu halten. Häufigste Komplikation nach Biopsie ist die Transplantatpankreatitis. In seltenen Fällen kann es zu Blutungen sowie Abszess- oder Fistelentstehungen kommen. Die Notwendigkeit eines chirurgischen Eingriffes als Folge einer perkutanen Biopsie ist insgesamt sehr selten [33, 39].

## Ergebnisse und Verlauf nach Pankreastransplantation (Tab. 3)

Im Vergleich zu den US-amerikanischen Spenderdaten sind deutsche Pankreasspender signifikant älter und der Anteil zerebrovaskulärer Todesursachen ist höher. Trotz dieser Tatsache konnten in den letzten Jahren sehr gute Ergebnisse nach PTX in deutschen Zentren erzielt werden [40]. Mit einem 1‑Jahres-Patienten- und 1‑Jahres-Transplantat-Überleben von 92 % und 83 % stehen diese den internationalen Ergebnissen in nichts nach (Tab. 3). Die Überlebensraten sind für die verschiedenen Formen der PTX nicht direkt vergleichbar, da es sich um Patientenkollektive mit unterschiedlicher Morbidität handelt (z. B. urämische vs. nichturämische Patienten). Die besten Ergebnisse werden dabei nach kombinierter SPK erzielt, mit einem Pankreastransplantatüberleben von 89 %, 71 % und 57 % nach 1, 5 und 10 Jahren. Für die gleichen Zeiträume liegen die Ergebnisse nach alleiniger PTA bei 84 %, 52 % und 38 % [2]. Die Transplantat-Halbwertszeiten für Pankreata (50 % Funktionsrate) werden mit 14 Jahren (SPK) und 7 Jahren (PAK, PTA) angegeben [2]. Durch die langfristige Normalisierung des Glukosestoffwechsels kommt es zu einer signifikanten Senkung der Mortalität, welche bei der SPK deutlich größer ist als bei alleiniger Nierentransplantation bei einem Typ-1-Diabetiker [8].

**Table 3:** 

QI/Bezeichnung	Referenzbereich (%)	2016/2017 (%)	2018/2019 (%)
Sterblichkeit im Krankenhaus	< 5	3,57	4,76
1‑Jahres-Überleben bei bekanntem Status	> 90	92,96	91,36
2‑Jahres-Überleben bei bekanntem Status	> 80	91,52	91,23
3‑Jahres-Überleben bei bekanntem Status	> 75	91,10	90,20
Qualität der Transplantatfunktion bei Entlassung	> 75	78,95	83,23
Qualität der Transplantatfunktion (1 Jahr nach Entlassung)	Nicht definiert	83,07	92,16
Qualität der Transplantatfunktion (2 Jahre nach Entlassung)	Nicht definiert	78,95	83,23
Qualität der Transplantatfunktion (3 Jahre nach Entlassung)	Nicht definiert	74,67	75,79

## Aufarbeitung und Beurteilung von Pankreas- und Duodenal-Abstoßungsbiopsien

Wie bei Organbiopsien üblich erfolgt nach Eingang und Prüfung der klinischen Angaben zunächst eine makroskopische Beurteilung der formalinfixierten Proben, wobei hier für eine erste Einschätzung auch ein Durchlichtmikroskop hilfreich sein kann. Da eine definitive Einordnung in endo- und exokrines Pankreas allerdings in der Lichtmikroskopie des Feuchtmaterials nicht möglich ist, empfiehlt sich zunächst eine Herstellung von HE- und PAS-Schnitten und die Anfertigung von mindestens 6 Leerschnitten für ergänzende histochemische und immunhistologische Untersuchungen, die nach Prüfung des Gewebes an den HE-/PAS-Schnitten angefordert werden können. Weiterhin empfiehlt es sich, in jedem Fall eine Bindegewebsfaserfärbung (z. B. Siriusrot, Elastica-van-Gieson [EvG] oder Mason-Goldner [MG]) und die 5 folgenden immunhistologischen Färbungen anzufertigen: C4d als Marker einer antikörpervermittelten Transplantatabstoßung (ABMR), CD3 als T‑Zell- und CD68 als Makrophagenmarker sowie Insulin und Glukagon zur Visualisierung der endokrinen Pankreasinseln [28, 38, 39].

Ein entsprechendes Vorgehen – allerdings primär ohne ergänzende immunhistologische Untersuchungen – wird auch für die selten durchgeführten und schwieriger zu beurteilenden Biopsien des Spenderduodenums durchgeführt, die hinsichtlich ihrer Signifikanz für die Pankreasabstoßung insgesamt sehr kontrovers diskutiert werden [41].

## Molekulare Marker der humoralen Pankreasabstoßung

Eine Analyse von 38 ausgewählten Genen (u. a. endotheliale Gene, NK-Zellgene und Entzündungsgene) mittels NanoString-nCounter-Technologie an 52 formalinfixierten Pankreasbiopsien zeigte, dass durch ergänzende molekulare Marker die Pankreasabstoßungsdiagnostik verbessert werden kann [42]. Diese aufwendige und kostenintensive molekulare Zusatzdiagnostik wird derzeit jedoch nicht routinemäßig eingesetzt.

## Häufige histomorphologische Befunde an Pankreastransplantatbiopsien (Abb. 4, 5, 6 und 7)

**Figure 4:**
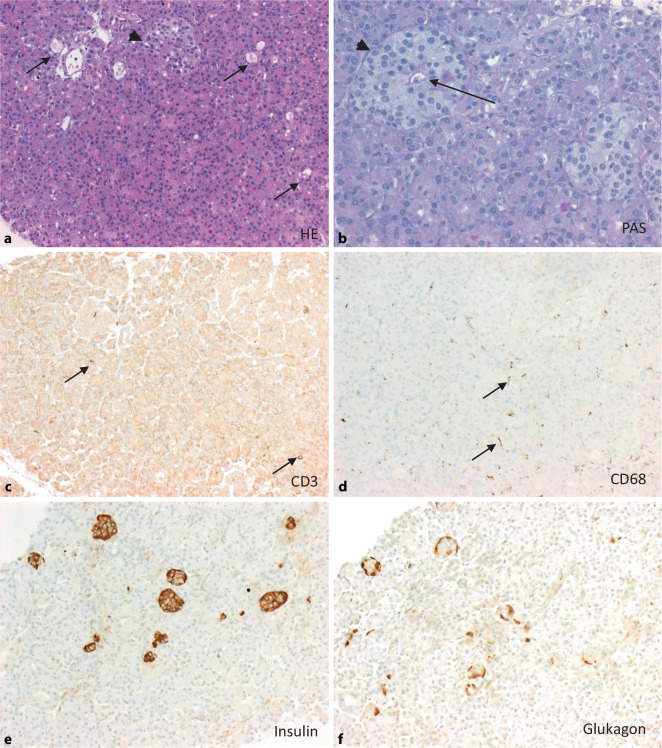

Für die Beurteilung von transplantatassoziierten Veränderungen ist primär die Kenntnis der normalen Anatomie des Pankreas und der Normalbefunde der verwendeten immunhistologischen Färbungen hilfreich (Abb. 4). Zunächst sollte in der HE- und PAS-Färbung (Abb. 4a, b) geprüft werden, ob exo- und endokrines Pankreasgewebe vorhanden ist und wieviele Läppchen erfasst sind. In der CD3- und der CD68-Färbung zeigen sich üblicherweise nur sehr wenige T‑Zellen und Gewebehistiozyten/Makrophagen (Abb. 4c, d). In der Insulin- und der Glukagon-Immunhistologie lassen sich die endokrinen Inseln und hier speziell die Alpha- und Betazellen meist sehr schön darstellen (Abb. 4e, f).

**Figure 5:**
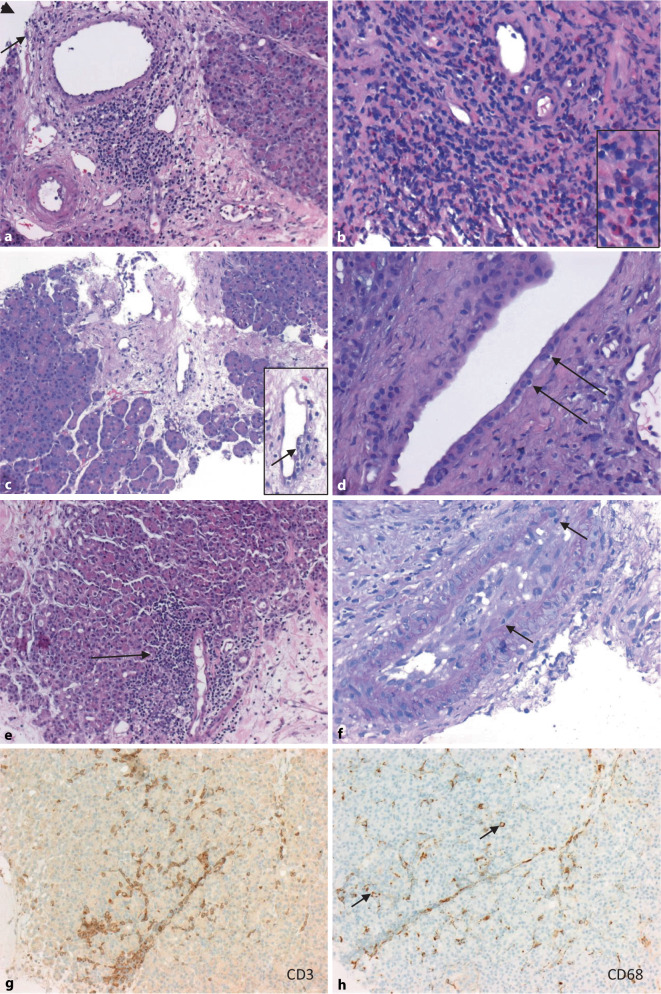

**Figure 6:**
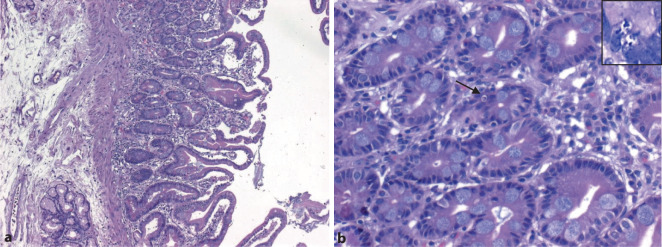

**Figure 7:**
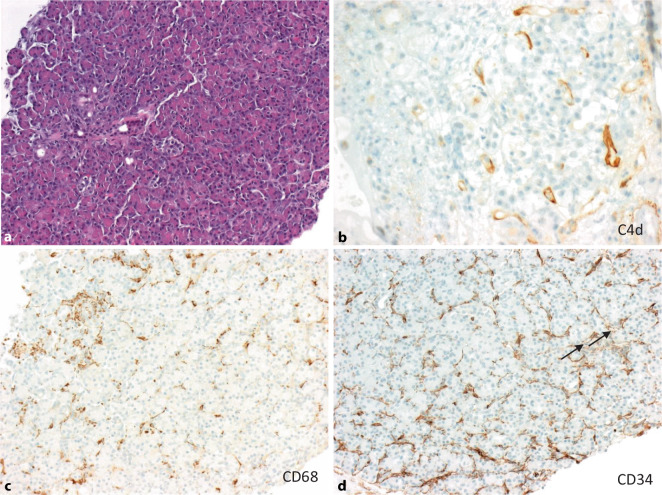

## Akute T-Zell- und akute antikörpervermittelte Transplantatabstoßung (Tab. 1, 4, 5 und 6)

Die morphologischen Veränderungen des Pankreas werden gemäß der aktuellen international gültigen Banff-Klassifikation eingeteilt [30]: Bei der häufigen akuten T‑Zell-vermittelten Transplantatabstoßung (TCMR; Tab. 1; Abb. 5) finden sich die folgenden histologischen Veränderungen: septale Entzündung mit aktivierten Lymphozyten- und z. T. Eosinophileninfiltraten, Venulitis und Duktitis, azinäre Entzündung und Endothelialitis (Abb. 5a–f). Das Ausmaß der T‑zellulären oder makrophagozytären Infiltration lässt sich hierbei gut durch ergänzende CD3- (Abb. 5g) bzw. CD68-Färbung (Abb. 5h) visualisieren, wobei hier v. a. das T‑zelluläre Infiltrat eine größere Rolle als das monozytär/makrophagozytäre Infiltrat spielt (Tab. 4). Die TCMR wird entsprechend ihres Schweregrades in 3 Kategorien (mild, moderat, schwer) eingeteilt, wobei die schwere Form mit diffuser azinärer Entzündung, fokaler oder diffuser Azinuszellnekrose und/oder moderater oder schwerer intimaler Arteriitis (> 25 % des Lumens) eine Abgrenzung zur akuten antikörpervermittelten Transplantatabstoßung (ABMR) notwendig macht, sodass hier in jedem Fall nach dem Vorhandensein donorspezifischer Antikörper (DSA) zu fragen ist (Tab. 1).

**Table 4:** 

	TCMR	ABMR
Septale Infiltrate	+++	− bis +
Eosinophile Granulozyten	+ bis +++	− bis +
Neutrophile Granulozyten	− bis ++	± bis +++
T‑Lymphozyten	++ bis +++	± bis +
Makrophagen	++	++++

**Table 5:** 

Grad	Wichtigste histologische Befunde
Nicht eindeutig bzw. ausreichend für eine TCMR	Minimales lokalisiertes Entzündungsinfiltrat, minimaler Kryptenepithelschaden, vermehrte epitheliale Apoptosen (< 6 Apoptosekörperchen/10 Krypten), keine oder nur minimale Architekturstörung der Schleimhaut, keine Schleimhautulzerationen und keine eindeutigen Veränderungen einer milden TCMR
Milde TCMR	Mildes lokalisiertes Entzündungsinfiltrat mit aktivierten Lymphozyten, milder Kryptenepithelschaden, vermehrte epitheliale Apoptosen (> 6 Apoptosekörperchen/10 Krypten), milde Architekturstörung der Schleimhaut, keine Schleimhautulzerationen
Moderate TCMR	Diffuses Entzündungsinfiltrat in der Lamina propria, diffuser Kryptenepithelschaden, vermehrt epitheliale Apoptosen mit fokaler Konfluenz, stärkere Schleimhautarchitekturstörung, milde bis mäßige intimale Arteriitis möglich, keine Schleimhautulzerationen
Schwere TCMR	Veränderungen der moderaten TCMR plus mukosale Ulzerationen bzw. auch schwere oder transmurale intimale Arteriitis möglich

*TCMR* T-Zell-vermittelte Abstoßung

**Table 6:** 

	Anzahl der Fälle (Prozentwert %)
*Alle Biopsien*^*a*^	
Pankreasbiopsien/Duodenalbiopsien	93 (100)/3
Kein Material (Pankreasbiopsien)	32 (34,4)
*Pankreasbiopsien mit verwertbarem Material*^*b*^	
Pankreasbiopsien gesamt	61 (100)
Keine Abstoßung	15 (24,6)
Akute TCMR	Indeterminate: 4 (6,6) Grad I: 30 (49,2) Grad II: 8 (13,1)
Aktive ABMR	Definitiv: 0 (0) Erwägen: 3 (4,9) Ausschließen: 2 (3,3)
Akuter Azinusepithelschaden	36 (59)
Allograftfibrose	Grad I: 29 (47,5) Grad II: 2 (3,3) Grad III: 2 (3,3)
Chronische Allograftarteriopathie	1 (1,6)
Insulitis	2 (möglich) (3,3)
CNI-Toxizität	3 (möglich) (4,9)
Pankreatitis	5 (8,2)

*ABMR* antikörpervermittelte Abstoßung, *CNI* Calcineurin-Inhibitor, *TCMR* T-Zell-vermittelte Abstoßung

^a^Prozentwerte bezogen auf alle Pankreasbiopsien unabhängig davon, ob verwertbares Material vorhanden war

^b^Prozentwerte bezogen auf Pankreasbiopsien mit verwertbarem Material

Auch im biopsierten Spenderduodenum (Tab. 5; Abb. 6a, b) können sich charakteristische Veränderungen einer TCMR mit unterschiedlich ausgeprägter Vermehrung von Lymphoyzten intraepithelial und in der Lamina propria sowie erhöhter epithelialer Apoptoserate finden sowie eine Architekturstörung der Schleimhaut (definiert als Verplumpung/Abflachung der Villi in dem am besten orientierten Schnitt) [43]. Im schweren Stadium zeigt sich auch eine ausgeprägte Schleimhautdestruktion mit Kryptenverlust, Verschorfung und neutrophilenreichem Infiltrat (Tab. 5; [43]). Hiervon abzugrenzen sind jedoch ischämische Veränderungen, die im Einzelfall ein ähnliches histologisches Bild induzieren können.

Die ABMR manifestiert sich am transplantierten Pankreas als mikrovaskulärer Endothelzellschaden des exokrinen Parenchyms, interazinäre Entzündung, Azinusepithelschaden, Vaskulitis und Thrombose, kann in der HE-Färbung aber auch relativ blande aussehen (Abb. 7a). Neben DSA und der Morphologie ist auch die spezifische C4d-Positivität der interazinären Kapillaren eines der 3 diagnostischen Kriterien der ABMR [29, 30], wofür eine C4d-Färbung notwendig ist (Abb. 7b). Im Gegensatz zur TCMR ist die ABMR v. a. durch ein monozytär/makrophagozytäres Infiltrat charakterisiert (Tab. 4; Abb. 7c). Zur Darstellung der interazinären Kapillaren und speziell zur Beurteilung der Dilatation und Endothelzellschwellung als Zeichen der kapillären Schädigung kann eine CD34-Immunhistochemie hilfreich sein (Abb. 7d).

## Nicht abstoßungsbedingte histomorphologische Veränderungen in Pankreasbiopsien nach Transplantation (Abb. 8)

**Figure 8:**
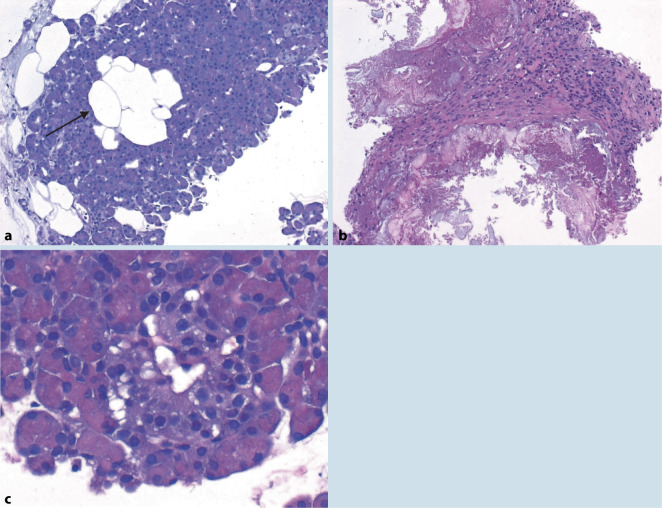

Hier sind zum einen anatomische bzw. nicht krankheitswertige Variationen, wie z. B. die relativ häufige Vermehrung von Fettzellen im Pankreas (Abb. 8a), [44] aber auch z. T. transplantationsassoziierte Veränderungen wie tryptische Pankreasnekrosen (Abb. 8b), ischämische Posttransplantationspankreatitis, Peripankreatitis bzw. peripankreatische Flüssigkeitsansammlung/Ödem, CMV-Pankreatitis, posttransplantations-lymphoproliferative Erkrankung (PTLD), bakterielle Infektionen oder Pilzinfektionen, Rekurrenz der Autoimmunerkrankung/des Diabetes mellitus oder eine CNI-Toxizität, die sich meist als Inselzellschaden, wie z. B. eine Vakuolisierung der endokrinen Zellen zeigt (Abb. 8c; [32]), zu nennen. Die Vakuolisierung von Inselzellen war in einer Studie, die die histologischen Befunde bei den beiden CNIs Cyclosporin A und Tacrolimus untersuchte, bei 2 Kontrollfällen nur minimal ausgeprägt und grobe Vakuolisierungen – wie bei CNI-Toxizität – wurden nicht beobachtet. Als weitere Merkmale der CNI-Toxizität fanden sich Inselzellschwellungen, Apoptosen und eine verminderte Reaktivität für Insulin in der Immunhistochemie [32].

## Erfahrungen der gemeinsamen PTX-Biopsiediagnostik Bochum-Erlangen (06/2017–12/2020, Tab. 6; Abb. 9)

**Figure 9:**
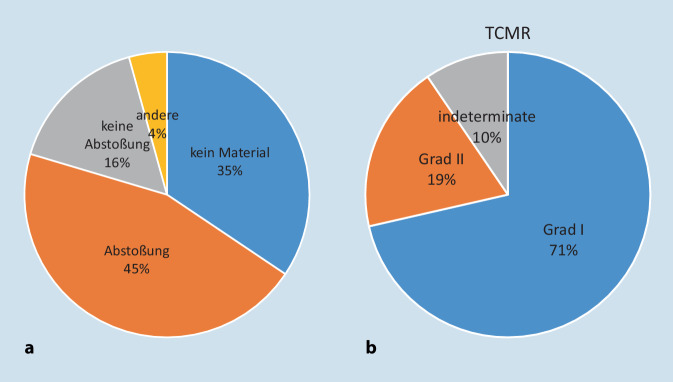

Im Zeitraum von Juni 2017 bis Dezember 2020 wurden aus der Chirurgischen Klinik des Universitätsklinikum Knappschaftskrankenhaus Bochum insgesamt 93 Pankreastransplantatbiopsien und 3 Duodenalbiopsien des Spenderduodenums von 49 Patienten in der Nephropathologie Erlangen untersucht. Die Ergebnisse der Pankreasbiopsien, wie in den Originalbefunden dokumentiert, sind in Tab. 6 zusammengefasst und in Abb. 9 teilweise illustriert. In einem Drittel der Biopsien (34,4 %) wurde kein diagnostisches Material gewonnen. Die bei weitem häufigste Diagnose war eine TCMR. Zur Einordnung der Befunde wie Insulitis und CNI-Toxizität oder einer ABMR ist immer der klinische Kontext von größter Wichtigkeit zur abschließenden Interpretation, sodass häufig nur anhand der Histologie eine definitive Diagnose nicht zu stellen ist. Im gleichen Zeitraum wurden 3 Biopsien des Spenderduodenums eingesandt, von denen eine Zeichen einer TCMR zeigte und 2 weitere keine Abstoßungszeichen.

## Fazit für die Praxis

- Die Biopsie des transplantierten Pankreas oder in seltenen Fällen auch des Spenderduodenums mit anschließender standardisierter Beurteilung entsprechend der aktuellen international gültigen Banff-Klassifikation der Pankreasabstoßung und der Empfehlungen zur Beurteilung von Duodenalbiopsien hat ihren festen Stellenwert in der Behandlung Pankreas‑/Nierentransplantierter Patienten.
- Wie bei anderen transplantierten parenchymatösen Organen werden die Biopsien nach klinischer Indikationsstellung durchgeführt und anschließend in entsprechend ausgerüsteten Pathologien gemäß der aktuell geltenden Einteilungen standardisiert aufgearbeitet und beurteilt.
- Diese Einordnung der morphologischen Veränderungen nach der Banff-Klassifikation der Pankreastransplantatabstoßung ist dann wiederum mit entsprechenden klinischen Handlungsanweisungen verknüpft, die in größeren klinischen Studien erprobt wurden und werden und mit entsprechenden Erfolgswahrscheinlichkeiten einhergehen.

## References

[1] Kelly WD, Lillehei RC, Merkel FK (1967). Allotransplantation of the pancreas and duodenum along with the kidney in diabetic nephropathy. Surgery.

[2] Gruessner AC, Gruessner RWG (2018). Pancreas transplantation for patients with type 1 and type 2 diabetes mellitus in the United States: a registry report. Gastroenterol Clin North Am.

[3] Land W, Illner WD, Abendroth D, Landgraf R (1984). Experience with 13 segmental pancreas transplants in cyclosporine-treated diabetic patients using ethibloc for duct obliteration (surgical aspects). Transplant Proc.

[4] Dholakia S, Royston E, Quiroga I (2017). The rise and potential fall of pancreas transplantation. Br Med Bull.

[5] Hanlon M, Cooper M, Abrams P (2019). Quality of life after pancreas transplantation: time to look again. Curr Opin Organ Transplant.

[6] Posegger KR, Linhares MM, Mucci S, Romano TM, Gonzalez AM, Salzedas Netto AA, Rangel ÉB, Lopes Filho GJ, Silva-Junior HT, Medina-Pestana J (2020). The quality of life in type I diabetic patients with end-stage kidney disease before and after simultaneous pancreas-kidney transplantation: a single-center prospective study. Transpl Int.

[7] Martins LS, Outerelo C, Malheiro J, Fonseca IM, Henriques AC, Dias LS, Rodrigues AS, Cabrita AM, Noronha IL (2015). Health-related quality of life may improve after transplantation in pancreas-kidney recipients. Clin Transplant.

[8] Morath C, Zeier M, Dohler B (2008). Metabolic control improves long-term renal allograft and patient survival in type 1 diabetes. J Am Soc Nephrol.

[9] Wai PY, Sollinger HW (2011). Long-term outcomes after simultaneous pancreas-kidney transplantation. Curr Opin Organ Transplant.

[10] Wullstein C, Woeste G, Taheri AS (2003). Morbidity following simultaneous pancreas/kidney transplantation. Chirurg.

[11] Deutsche Stiftung Organtransplantation Tätigkeitsbericht Pankreastransplantation 2019. https://www.dso.de/BerichteTransplantationszentren/Grafiken%20D%202019%20Pankreas.pdf. Zugegriffen: 5.1.2021

[12] Perosa M, Boggi U, Cantarovich D (2011). Pancreas transplantation outside the USA: an update. Curr Opin Organ Transplant.

[13] Landgraf R, Dieterle C (2010). Pankreasorgantransplantation bei Typ-1-Diabetes-Patienten. Diabetologe.

[14] Gruessner AC, Sutherland DE, Dunn DL (2001). Pancreas after kidney transplants in posturemic patients with type I diabetes mellitus. J Am Soc Nephrol.

[15] Bundesärztekammer Richtlinien. http://www.bundesaerztekammer.de/fileadmin/user_upload/downloads/pdf-Ordner/RL/RiliOrgaWlOvPankreasTx20200714.pdf10.3238/arztebl.2020.rili_baek_OrgaWlOvPankreasTx20200714. Zugegriffen: 5.1.2021

[16] Sharples EJ, Mittal SM, Friend PJ (2016). Challenges in pancreas transplantation. Acta Diabetol.

[17] Schenker P, Vonend O, Krüger B (2011). Long-term results of pancreas transplantation in patients older than 50 years. Transpl Int.

[18] Scalea JR, Redfield RR, Arpali E (2016). Pancreas transplantation in older patients is safe, but patient selection is paramount. Transpl Int.

[19] Fridell JA, Rogers J, Stratta RJ (2010). The pancreas allograft donor: current status, controversies, and challenges for the future. Clin Transplant.

[20] Muthusamy ASR, Vaidya A (2011). Expanding the donor pool in pancreas transplantation. Curr Opin Organ Transplant.

[21] Eurotransplant International Foundation Annual report 2019. https://www.eurotransplant.org/wp-content/uploads/2020/06/Annual-Report-2019.pdf. Zugegriffen: 5.1.2021

[22] Sollinger HW, Cook K, Kamps D, Glass NR, Belzer FO (1984). Clinical and experimental experience with pancreaticocystostomy for exocrine pancreatic drainage in pancreas transplantation. Transplant Proc.

[23] Nghiem DD, Corry RJ (1987). Technique of simultaneous renal pancreatoduodenal transplantation with urinary drainage of pancreatic secretion. Am J Surg.

[24] Boggi U, Vistoli F, Signori S (2005). A technique for retroperitoneal pancreas transplantation with portal-enteric drainage. Transplantation.

[25] Walter M, Jazra M, Kykalos S (2014). 125 Cases of duodenoduodenostomy in pancreas transplantation: a single-centre experience of an alternative enteric drainage. Transpl Int.

[26] Uva PD, Papadimitriou JC, Drachenberg CB (2019). Graft dysfunction in simultaneous pancreas kidney transplantation (SPK): Results of concurrent kidney and pancreas allograft biopsies. Am J Transplant.

[27] Parajuli S, Arpali E, Astor BC (2018). Concurrent biopsies of both grafts in recipients of simultaneous pancreas and kidney demonstrate high rates of discordance for rejection as well as discordance in type of rejection—a retrospective study. Transpl Int.

[28] Drachenberg CB, Odorico J, Demetris AJ (2008). Banff schema for grading pancreas allograft rejection: working proposal by a multi-disciplinary international consensus panel. Am J Transplant.

[29] Drachenberg CB, Torrealba JR, Nankivell BJ, Rangel EB, Bajema IM, Kim DU (2011). Guidelines for the diagnosis of antibody-mediated rejection in pancreas allografts-updated Banff grading schema. Am J Transplant.

[30] Loupy A, Haas M, Solez K, Racusen L, Glotz D, Seron D (2017). The Banff 2015 kidney meeting report: current challenges in rejection classification and prospects for adopting molecular pathology. Am J Transplant.

[31] Redfield RR, Kaufmann DB, Odorico JS (2015). Diagnosis and treatment of pancreas rejection. Curr Transplant Rep.

[32] Drachenberg CB, Klassen DK, Weir MR, Wiland A, Fink JC, Bartlett ST, Cangro CB, Blahut S, Papadimitriou JC (1999). Islet cell damage associated with tacrolimus and cyclosporine: morphological features in pancreas allograft biopsies and clinical correlation. Transplantation.

[33] Gaber LW (2007). Pancreas allograft biopsies in the management of pancreas transplant recipients: histopathologic review and clinical correlations. Arch Pathol Lab Med.

[34] Uva PD, Odorico JS, Giunippero A, Cabrera IC, Gallo A, Leon LR, Minue E, Toniolo F, Gonzalez I, Chuluyan E, Casadei DH (2017). Laparoscopic biopsies in pancreas transplantation. Am J Transplant.

[35] Nakhleh RE, Benedetti E, Gruessner A, Troppmann C, Goswitz JJ, Sutherland DE, Gruessner RW (1995). Cystoscopic biopsies in pancreaticoduodenal transplantation. Are duodenal biopsies indicative of pancreas dysfunction?. Transplantation.

[36] Nordheim E, Horneland R, Aandahl EM (2018). Pancreas transplant rejection episodes are not revealed by biopsies of the donor duodenum in a prospective study with paired biopsies. Am J Transplant.

[37] Margreiter C, Aigner F, Resch T, Berenji AK, Oberhuber R, Sucher R, Profanter C, Veits L, Öllinger R, Margreiter R, Pratschke J, Mark W (2012). Enteroscopic biopsies in the management of pancreas transplants: a proof of concept study for a novel monitoring tool. Transplantation.

[38] Wan J, Fang J, Li G, Xu L, Yin W, Xiong Y, Liu L, Zhang T, Wu J, Guo Y, Ma J, Chen Z (2019). Pancreas allograft biopsies procedure in the management of pancreas transplant recipients. Gland Surg.

[39] Klassen DK, Weir MR, Cangro CB (2002). Pancreas allograft biopsy: safety of percutaneous biopsy-results of a large experience. Transplantation.

[40] Institut für Qualitätssicherung und Transparenz im Gesundheitswesen QS-Berichte. https://iqtig.org/downloads/auswertung/2019/pntx/QSKH_PNTX_2019_BUAW_V02_2020-07-14.pdf. Zugegriffen: 5.1.2021

[41] Drachenberg CB (2019). Is the duodenum trustworthy?. Transplantation.

[42] Roufosse C, Drachenberg C, Renaudin K, Willicombe M, Toulza F, Dominy K, McLean A, Simmonds N, de Kort H, Cantarovitch D, Scalea J, Mengel M, Adam B (2020). Molecular assessment of antibody-mediated rejection in human pancreas allograft biopsies. Clin Transplant.

[43] Wu T, Abu-Elmagd K, Bond G, Nalesnik MA, Randhawa P, Demetris AJ (2003). A schema for histologic grading of small intestine allograft acute rejection. Transplantation.

[44] Majumder S, Philip NA, Takahashi N, Levy MJ, Singh VP, Fatty Pancreas CST (2017). Should We Be Concerned?. Pancreas.

